# Effects of a digital self-efficacy training in stressed university students: A randomized controlled trial

**DOI:** 10.1371/journal.pone.0305103

**Published:** 2024-10-31

**Authors:** Judith Rohde, Marta A. Marciniak, Mirka Henninger, Stephanie Homan, Anja Ries, Christina Paersch, Olivia Friedman, Adam D. Brown, Birgit Kleim

**Affiliations:** 1 Department of Adult Psychiatry and Psychotherapy, Psychiatric University Hospital Zurich, Zurich, Switzerland; 2 Department of Psychology, University of Zurich, Zurich, Switzerland; 3 Department of Psychology, New School for Social Research, New York, New York, United States of America; 4 Department of Psychiatry, New York University School of Medicine, New York, New York, United States of America; Northern Arizona University, UNITED STATES

## Abstract

**Objective:**

Self-efficacy is associated with positive mental health outcomes. We developed and tested a digital self-efficacy training for daily recall of autobiographical self-efficacy memories (e.g., memories of successfully overcoming a personal challenge).

**Method:**

In this randomized controlled trial, we investigated the effects of the week-long digital self-efficacy training on key mental health outcomes, including anxiety, stress, and hopelessness, and on self-efficacy in 93 university students (mean age 23.3 years, *SD*: 3.49) with elevated self-reported stress levels. Participants completed either the self-efficacy training combined with ecological momentary assessment (EMA) (training group) or EMA only (control group).

**Results:**

We found significantly reduced hopelessness and trait anxiety in the training group compared to the control group at post-assessment (one day post intervention). Effects on ratings of self-efficacy at post-assessment were also significant when controlling for baseline self-efficacy.

**Conclusions:**

This stand-alone digital self-efficacy training was significantly associated with a number of positive effects on outcomes compared to a control condition, including reduced hopelessness, trait anxiety, and increased self-efficacy. Future work is needed to replicate and investigate the long-term effects of the training and explore its implementation in clinical populations.

**Trial registration:**

ClinicalTrials.gov Identifier: NCT05617248

## Introduction

Self-efficacy refers to an individual’s belief in their ability to exercise control over their environment, thoughts, emotions, and behavior and has been associated with positive mental health outcomes and adaptation to adverse events [1, 2]. In terms of mechanisms, self-efficacy was associated with being better able to manage social and professional challenges through its effects on positive motivation and the effort individuals will exert to achieve desired outcomes [3, 4]. Self-efficacy beliefs (e.g., a sense of mastery, confidence in overcoming challenges) may act as a protective mechanism against psychological distress and support positive adaptation during times of uncertainty and adversity, such as during a pandemic [5–8]. Self-efficacy was associated with stress resilience [9] and may act as a daily buffer against stress [9, 10].

University students are a particularly vulnerable group known to experience elevated levels of stress in different areas of life [11, 12], which has been found to be associated with reduced academic achievements and an elevated risk of school dropout [12]. Furthermore, the extent of stress is associated with increased odds of mental disorders [11]. However, high levels of self-efficacy in university students, for instance, positively affected academic performance and ability to cope with challenges in university [13, 14]. Similarly, high self-efficacy has been associated with reduced anxiety in a distressed student population [15] and it has been proposed as a protective factor for risk of persistent or recurrent major depressive disorders in students [16]. Also, positive relations between self-efficacy and hope [17, 18], self-efficacy and life satisfaction, and positive affect as well as an inverse relationship between self-efficacy and negative affect were reported [19].

Self-efficacy can be experimentally induced, which may result in clinically relevant improvements in cognitive, affective, and decision-making processes. Several studies employed false feedback induction, whereby non-clinical participants were led to believe they had high or low levels of self-efficacy. Individuals who received false feedback indicating high self-efficacy performed better on social problem-solving and autobiographical memory tasks [20]. In addition to the false feedback technique, autobiographical memory-based strategies have been used in some studies to increase self-efficacy. These studies propose that self-efficacy beliefs are construed, in part, from the internalization of successfully having overcome difficult life episodes (mastery experiences) [2]. Successful induction of mastery-related autobiographical memories has been associated, for instance, with a greater capacity to reappraise negative events, as documented by less distress when recalling negative personal memories [21]. It has also been shown that self-efficacy induction is associated with improved future thinking and decision-making [22]. Applying this memory-based technique to clinical populations was linked to neural changes in brain regions associated with executive function and emotion regulation compared to a control group that did not recall such memories [23]. Another study provided a self-efficacy skills training consisting of four 90-minute training sessions including lectures, role plays, and other content leading to significant improvements in self-efficacy in primary care health workers [24].

While previous research mostly focused on individual and single-session self-efficacy interventions delivered in the lab and face-to-face settings, mobile health interventions, which are delivered via mobile devices, may hold promise for modulating self-efficacy in individuals’ everyday lives. They offer a resource-efficient way of delivering mental health interventions repeatedly and whenever needed [25–29]. In contrast to mobile interventions, digital interventions could also be delivered via a computer. Mobile interventions may be useful in prevention settings and when therapy access is limited, such as in times of crises when psychological needs are heightened and therapist resources scarce [30–33]. For instance, Ecological Momentary Interventions (EMI) conveniently deliver scalable psychological interventions that fit well into the user’s daily routines. EMIs seem to increase their efficacy when combined with, e.g., Ecological Momentary Assessments (EMA) [34], which are mobile phone-based methods to measure transient parameters such as mood, activity, and within-person dynamics in the moment of life [35]. Promisingly, one study recently applied a brief digital psychotherapeutic intervention asking participants to record three good things each day, which was associated with significant improvements in job performance and self-efficacy after participation in the intervention group compared to the control group, which did not engage in any task [36]. Additionally, one recent study asked participants to recall self-efficacious memories digitally, which was associated with less covid-19-related fear [37]. In a mobile mental health intervention, self-efficacy mediated the effects of a digital psychotherapy program for mild to moderate depression on stress and anxiety symptoms. However, in that study, self-efficacy was not targeted directly [38]. The current study addresses this gap by conducting an experiment to enhance self-efficacy in a similarly accessible and cost-effective manner. By doing so, this study aims to inform future efforts centered on self-efficacy to holistically improve academic, professional, and social performance, as well as mental health, among college students.

We developed an EMI providing a digital self-efficacy training focusing on repeated recall of idiographic mastery experiences. In addition, we applied EMA daily measuring mood and social context. We investigated training effects in university students reporting elevated levels of stress and compared them with an active control also engaging in a digital application that EMA alone. We hypothesized that this digital self-efficacy training combined with EMA over the course of 7 days would result in higher self-efficacy in distressed university students and based on previous findings on associations of self-efficacy with other mental health aspects, greater improvements in anxiety, stress, hopelessness, hope, and positive and negative affect, compared to EMA alone. Therefore, our main research questions were to investigate the training’s effects on the primary outcome self-efficacy and on the secondary outcomes anxiety, stress, hopelessness, hope, and positive and negative affect (pre-post changes). Based on results from recent digital training studies mentioned above, we expected between-person variability in compliance and the proposed outcomes. We thus also investigated which baseline variables lead to the training being more effective, specifically regarding self-efficacy increase.

## Methods

### Design

**Fig 1:**
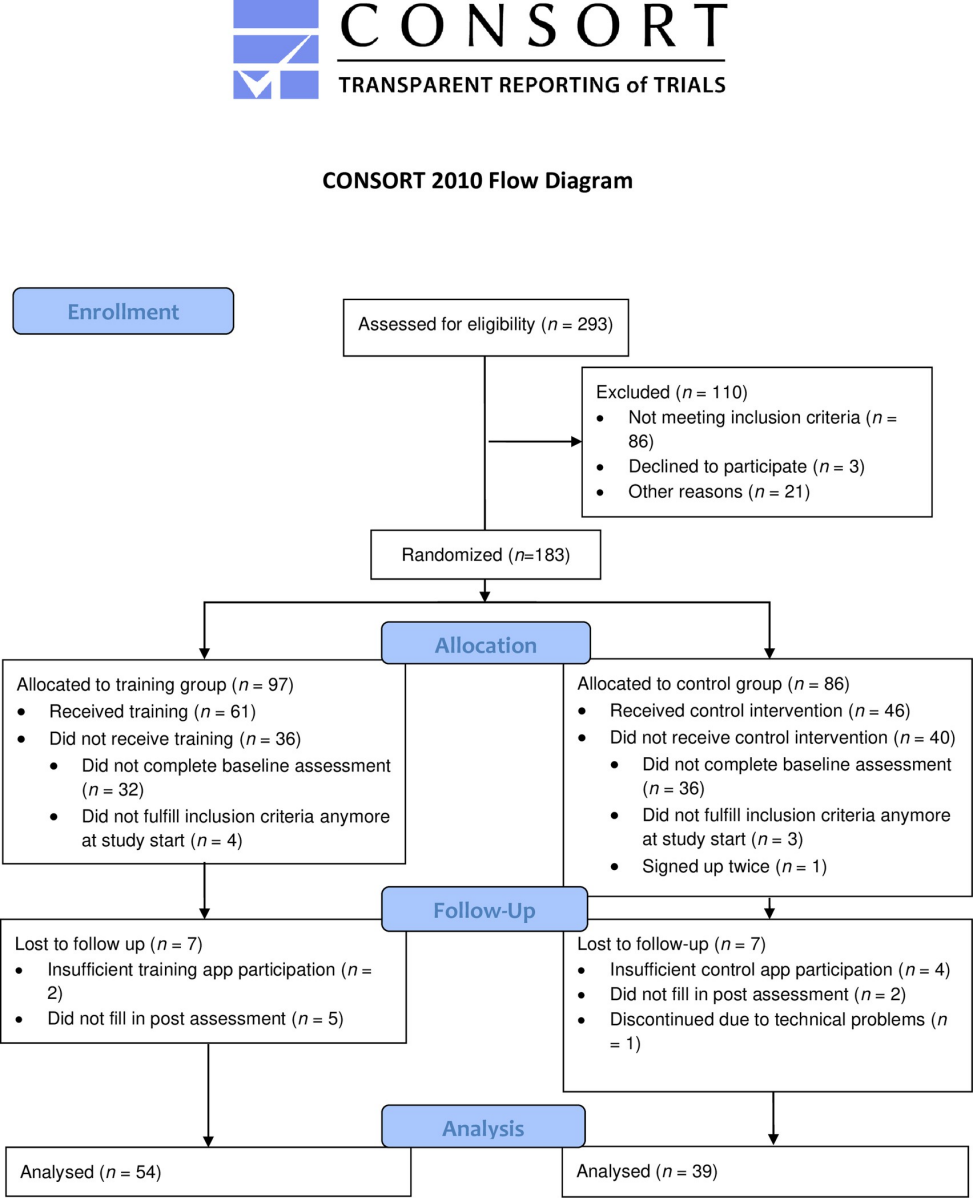
CONSORT flow diagram.

**Fig 2:**
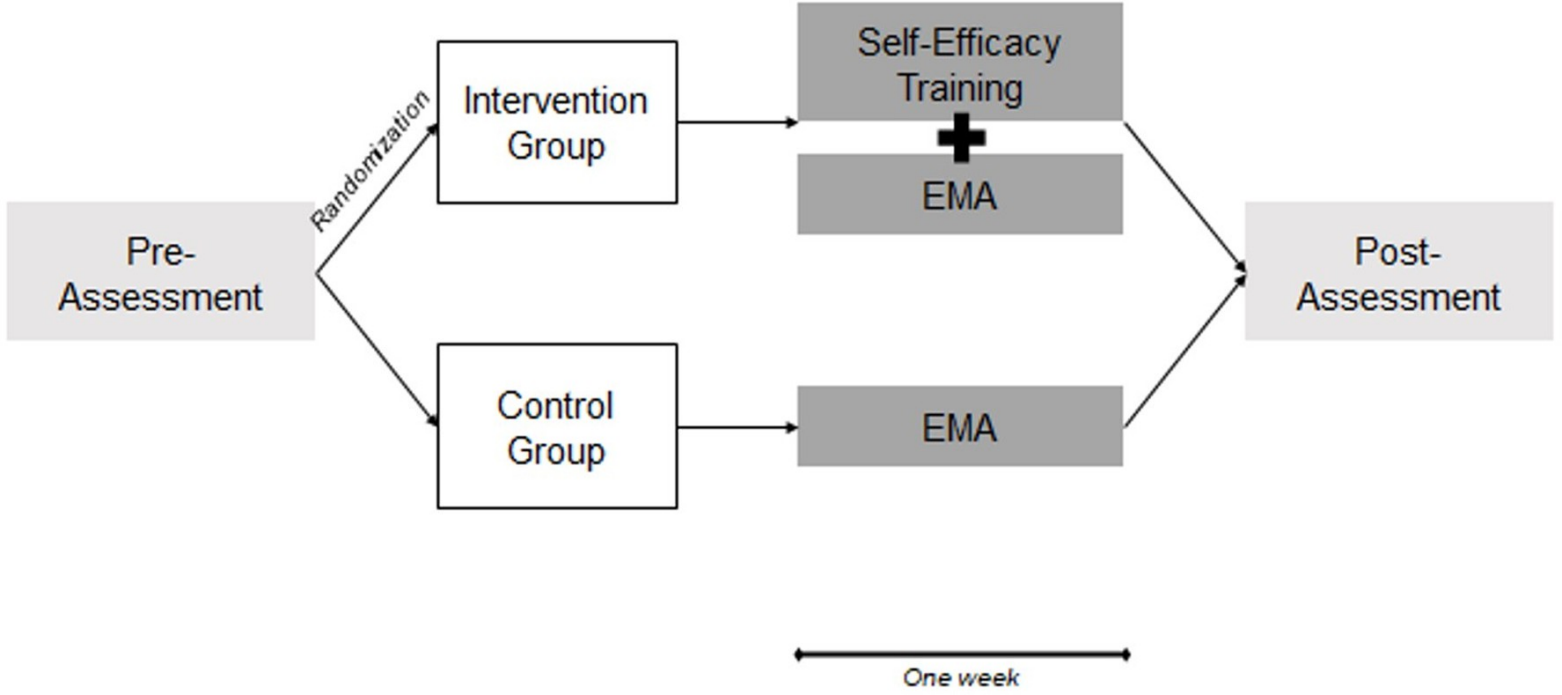
Study timeline and assessments.

The study was a randomized controlled trial (Fig 1). Ethical approval was obtained from the University of Zurich Ethics Committee of the Faculty of Arts (No. 20.4.24). The authors assert that all procedures contributing to this work comply with the ethical standards of the relevant national and institutional committees on human experimentation and with the Helsinki Declaration, as revised in 2013. Following piloting, some adaptations were made to the study protocol (study protocol and deviations can be found in the supporting information, see S2 and S3 Files). Following an eligibility screening, participants were randomly assigned to the training group or the control group. Independent researchers conducted random allocation. The random allocation sequence was generated by a software. The training group received a smartphone-based self-efficacy training combined with EMA, which was adapted from Marciniak and colleagues [39], while the control group received EMA only (Fig 2). Both the self-efficacy training and the EMA were administered through the SEMA3 platform, an open-source software application for the delivery of advanced smartphone surveys [40]. Participants completed baseline and post-assessments online. All data was collected digitally. This trial studying healthy subjects was not registered prior to beginning the project, but retrospectively (ClinicalTrials.gov Identifier: NCT05617248). The authors confirm that all ongoing and related trials for this intervention are registered. Our primary outcome was self-efficacy (changes from baseline to post-assessment). Secondary outcomes were anxiety, hopelessness, hope, stress, and positive and negative affect (changes from baseline to post-assessment).

### Participants

Participants were recruited from Swiss universities through online postings and mailing lists during the first and second wave of the COVID-19 pandemic (range for participant recruitment and follow-up: 05/2020–11/2020). They received a link to the study information, which provided adequate and well comprehensible information on the study and its procedures. Potential subjects were also provided with contact information of the study personnel and had the opportunity to contact them if there were any questions at this or a later time point. All participants provided electronic informed consent and were then able to fill in the screening questionnaire. The screening questionnaire addressed the inclusion and exclusion criteria. Participants were offered either a sum of up to 60 Swiss Francs (equivalent to US $65 at the time the study was conducted), depending on compliance, or course credits. Participants with a current psychiatric disorder were excluded. The inclusion criteria were enrolment at a Swiss university, aged between 18 and 29 years, owning a smartphone, speaking fluent German, and experiencing heightened stress indicated by a score of ≥13 on the Perceived Stress Scale (PSS), consisting of 10 items with five possible answers (0–4) with higher sum scores indicating greater levels of perceived stress [41–44]. The PSS showed a good internal consistency of *α* = 0.84 in a representative sample using the German version [41] and at least acceptable test-retest reliability with correlation coefficients assessed in different studies of between >0.70 [43] and 0.91 [44]. In our sample, the internal consistency was good (baseline: *α* = 0.83, post: *α* = 0.83), and.

Following the screening for inclusion and exclusion criteria, 183 individuals were randomly assigned to the training (*n* = 97) and control groups (*n* = 86). Participants who did not complete the baseline assessments (*n* = 32 in the training group, *n* = 36 in the control group) did not receive the self-efficacy training or, respectively, the EMA. Additionally, eight participants (*n* = 4 in the training group, *n* = 4 in the control group) were excluded because their situation had changed at this time point and they fulfilled diagnostic criteria for a psychiatric disorder, were no longer enrolled at a Swiss university at the start of the study or had applied for the study twice. This led to *n* = 61 participants receiving the self-efficacy training and *n* = 46 participants receiving the control intervention. Since only participants who actively responded to the app’s prompts (response rate >25%) and completed the post assessments were included in our analyses, seven participants in each group were lost to follow-up. The final sample consisted of 93 participants (*n* = 54 in the training group, *n* = 39 in the control group), 78% of whom were female. The mean age was 23.3 years (*SD*: 3.49). Table 1 summarizes the demographic and clinical variables. Table 2 provides additional demographic information.

**Table 1:** Demographic and clinical variables and group differences.

Variable	Training group n = 54			Control group n = 39			Group difference
	*n* (%)	*M*	*SE*	*n* (%)	*M*	*SE*	*p-value* *
**Female sex**	42 (77.78)			31 (79.49)			.84
**Age**		23.72	0.44		22.64	0.61	.14
**Perceived stress (PSS)**		2.25	0.081		2.13	0.095	.34
**Depressive symptoms (BDI-II)**		0.67	0.059		0.55	0.072	.22
**State anxiety (STAI, state subscale)**		2.18	0.078		2.12	0.087	.60
**Trait anxiety (STAI, trait subscale)**		2.27	0.079		2.08	0.072	.085
**Positive affect (PANAS, positive affect subscale)**		2.64	0.10		2.71	0.12	.66
**Negative affect (PANAS, negative affect subscale)**		1.69	0.093		1.67	0.089	.89
**Hopelessness (BHS)**		0.28	0.031		0.21	0.028	.11
**Self-efficacy (GSE)**		2.69	0.057		2.79	0.073	.30

SE = Standard Error; PSS = Perceived Stress Scale; BDI-II = Beck Depression Inventory; STAI = State and Trait Anxiety Scale; PANAS = Positive and Negative Affect Schedule; BHS = Beck Hopelessness Scale; GSE = General Self-Efficacy Scale

* χ^2^-test for categorical data and independent samples t-test for continuous data

**Table 2:** Demographic variables.

Education	Percentage (total number)
Master’s degree	10% (*n* = 9)
Bachelor’s degree	41% (*n* = 38)
Other	49% (*n* = 46)
**Major**	
Psychology	40% (*n* = 37)
Law	9% (*n* = 8)
Natural science	8% (*n* = 7)
Engineering	5% (*n* = 5)
Other	62% (*n* = 36)
**Origin**	
Switzerland	80% (*n* = 74)
Germany	11% (*n* = 10)
Other European countries	8% (*n* = 7)
Non-European countries	2% (*n* = 2)

### Procedure and measures

#### Screening

Individuals completed the German version of the PSS and provided basic demographic information.

#### Baseline assessment

Participants were asked to complete an online baseline assessment. If they did not complete the assessment within one week, they were reminded by e-mail.

The baseline assessment consisted of the following self‐report questionnaires (German versions), which have been psychometrically tested and proved reliability and validity:

1. Beck Depression Inventory-II (BDI-II): This questionnaire includes 21 items with four possible answers (0–3) measuring depression severity, e.g., with a score of 10–18 indicating mild depression. It provides good to excellent internal consistency (*α* ≥ 0.84 in a German sample and 0.90 across various) and a test-retest reliability of r ≥ 0.75 ranging from 0.73 to 0.96) [45–47]. In our sample, the internal consistency was good as well (baseline: *α* = 0.90, post: *α* = 0.88).
2. State-Trait Anxiety Inventory (STAI): This instrument consists of 40 items on a 4-point Likert Scale, with a commonly used cutoff score of 40 indicating probable clinical levels of anxiety. It has high internal consistency (α of around 0.90) and high test-retest reliability for the trait subscale (ranging from 0.73 to 0.90). The test-retest reliability for the state subscale is lower as expected from the concept’s definition [48–51];). The internal consistency in our sample was high (state subscale: baseline: *α* = 0.92, post: *α* = 0.91; trait subscale: baseline: *α* = 0.92, post: *α* = 0.90).
3. Positive and Negative Affect Scale (PANAS): The scale consists of 20 items with 10 items each measuring positive affect and negative affect using a 5-point Likert scale. Higher scores indicate greater levels of positive and negative affect, respectively [52–54]. The scale has good internal consistency (*α* ≥ 0.84, German version [53]). The test-retest reliability depends on the length of the rated time frame and increases if the time frame lengthens (between 0.54 and 0.68 for PANAS positive affect subscale and between 0.45 and 0.71 for PANAS negative affect subscale) [52–54] ;). The internal consistency in our sample was good (PANAS positive affect subscale: baseline: *α* = 0.87, post: *α* = 0.90; PANAS negative affect subscale: baseline: *α* = 0.86, post: *α* = 0.86).
4. Beck Hopelessness Scale (BHS). This questionnaire consists of 20 dichotomous true and false items assessing aspects of hopelessness, with higher scores indicating greater levels of hopelessness [55–57]. It has good internal consistency, with *α* = 0.87 in a representative German sample [56] and 0.93 in a clinical sample using the original version [55], and a good test-retest reliability of *r* = 0.81 [57];. The internal consistency in our sample was good (baseline: *α* = 0.86, post: *α* = 0.82).
5. General Self-Efficacy Scale (GSE): This scale consists of 10 items using a 4-point Likert Scale, assessing a general sense of perceived self-efficacy. Higher scores indicate greater levels of self-efficacy [58–62]. The scale has internal consistencies ranging from *α* = 0.75 to *α* = 0.94 [58, 61], and a test-retest reliability ranging from *r* = 0.47 to *r* = 0.75, depending on the sample and time frame [62]. Our sample showed good internal consistency (baseline: *α* = 0.84, post: *α* = 0.88).
6. Trait Hope Scale (THS): This instrument contains 12 items using an 8-point scale, with higher scores indicating greater hope [63–66]. It provides good internal consistency, with Cronbach’s alpha ranging from 0.74 to 0.88, and a test-retest reliability of 0.73 to 0.85 [63, 65, 66] (;). We found a good internal consistency in our sample (baseline: *α* = 0.86, post: *α* = 0.86).

#### Smartphone-based self-efficacy training

Participants of the training group received psychoeducation on self-efficacy, delivered via a 4:22 minute video, which is available on https://www.youtube.com/watch?v=NOMMb5sB9SE and were instructed to define two autobiographical memories of mastery experiences. They were asked to recall difficult situations they had managed successfully and were given examples. Regarding memory content, the most frequently reported memories were (in descending order) *academic successes* (e.g., passing an examination), *overcoming social challenges* (e.g., performances in front of an audience, losing weight), and *excelling in a sports event*. Subsequently, participants were sent installation instructions and a download link. After they had downloaded the self-efficacy app to their smartphones, they were able to access information about the study, app usage, and self-efficacy. Additionally, they were asked to record the two autobiographical memories they had been asked to think of. Specifically, they were asked to think of a title of their memories and then to briefly describe the situations. The self-efficacy training, which started the following day, was based on the previously defined autobiographical memories. During an imagination task, participants were instructed to perform a slow breathing technique and to imagine one of the memories step by step, focusing specifically on the character traits and qualities necessary to achieve what they had achieved. Participants received three self-efficacy training sessions per day. They were prompted within fixed 30-minute intervals, and the training sessions were no longer accessible if not reacted to within 50 minutes. Extra training sessions with EMA questions were available at any time and could be easily accessed using a button in the app The extra sessions provided the same content as the ones appearing at the prompted times except for the fact that the participant was asked to choose the memory they wanted to do the training with. On the first day, all trainings focused on memory one, on the second day all trainings focused on memory two, and so on. On the last day, participants could choose which memory they wanted to work with. Every evening, the training group received additional questions on specific self-efficacy, the analysis of which is not part of this paper. Table 3 provides an overview of the self-efficacy training procedures.

**Table 3:** Overview of self-efficacy training.

**Before app participation**	Psychoeducation video
	Definition of two autobiographical self-efficacy memories
	Receiving app download information, downloading app
**During app participation (one week)**	3x/day: Recall of self-efficacy memories
	10x/day: EMA
	Information about study, app usage, and self-efficacy as well as additional trainings and EMA were accessible at all time

#### Ecological momentary assessment

Both groups received EMA questions 10 times per day for seven days measuring mood, social contacts, and virtual context. For example, as part of the mood questions, participants were asked to what extent they agreed with statements such as “I feel cheerful”, “I feel irritated” etc. using a scale from 1 (not at all) to 7 (very much). All EMA questions are made available online. For the training group, three of the 10 daily question sets were combined with the self-efficacy training. Participants received the prompts at fixed times during the day. In addition to the scheduled questionnaires, participants could respond to additional EMA questions at any time, which could be easily accessed using a button in the app. The EMA questions were displayed in combination with the self-efficacy training if the participant was in the training group or alone if the participant was in the control group. The EMA data will be analyzed as part of another publication.

#### Post-assessment

The day after the one-week study period, participants received a link to the post-assessment, which was the same as the baseline assessment but also included the PSS and the German version of the user version of the Mobile Application Rating Scale, consisting of 20 items assessing engagement, functionality, esthetics, information, and subjective quality of mobile apps (uMARS; [67, 68]). Participants were asked to complete the assessment within 3 days.

#### Compliance

We defined compliance as the total number of push notifications responded to out of scheduled push notifications plus extra self-efficacy training sessions (training group) or EMA (control group). A compliance rating of 1 indicated the completion of all 70 scheduled tasks and a rate higher than 1 the completion of extra tasks. Looking at the groups individually, we found a mean compliance rate of 0.72 (*SD*: 0.18) in the training group and of 0.79 (*SD*: 0.25) in the control group. Across both groups, the compliance variable ranged from 0.28 to 1.46.

#### Blinding

Participants were blind to group assignment. The study information only contained the information that they were asked to participate in a one-week smartphone app daily assessing thoughts and emotions, which is applicable to both the training and control group. Study personnel could not be blinded, but emails and online assessments were standardized.

### Statistical analysis

All analyses were conducted in R 4.0.2 [69] and Mplus 8.6 [70] using the MplusAutomation package [71] and SPSS (Version 27.0, IBM SPSS Statistics, Switzerland). Data and all analyses were made publicly available and can be found on OSF (https://osf.io/ucqjk/?view_only=44948d16457b4d278baefe2f986ca940). All 93 participants were included in the analyses. Participants could only save their responses if they were complete. Therefore, there are no missing responses in the assessment. We visually inspected the distribution of the difference scores between the post and baseline assessment that can be found in the supporting information (S1 File).

We fixed the alpha error rate to α = .05 in all analyses. The effects of the self-efficacy training were investigated in two steps. First, we used a multivariate linear model in which we regressed the difference scores of the outcomes self-efficacy, hopelessness, state and trait anxiety, perceived stress, positive and negative affect, and hope on group and compliance to examine the effects of the self-efficacy training. The multivariate model allowed us to examine all outcome variables within a single model and to account for associations between the outcomes. The value *b* stands for the unstandardized regression coefficient. The regression equations are thus specified as follows:

$$BHS_{i}^{diff}=b_{0}+b_{1}group_{i}+b_{2}compliance_{i}$$

$$STAI-Trait_{i}^{diff}=b_{0}+b_{1}group_{i}+b_{2}compliance_{i}$$

$$STAI-State_{i}^{diff}=b_{0}+b_{1}group_{i}+b_{2}compliance_{i}$$

$$PSS_{i}^{diff}=b_{0}+b_{1}group_{i}+b_{2}compliance_{i}$$

$$Panas-Positive_{i}^{diff}=b_{0}+b_{1}group_{i}+b_{2}compliance_{i}$$

$$Panas-Negative_{i}^{diff}=b_{0}+b_{1}group_{i}+b_{2}compliance_{i}$$

$$GSE_{i}^{diff}=b_{0}+b_{1}group_{i}+b_{2}compliance_{i}$$

$$THS_{i}^{diff}=b_{0}+b_{1}group_{i}+b_{2}compliance_{i}$$

In the model, we also allowed the outcomes to be correlated with each other to account for their association in model estimation.

Second, we used a multiple linear regression model to investigate the interaction effects of baseline self-efficacy with hopelessness, hope, state and trait anxiety, positive and negative affect, depression, and perceived stress on the self-efficacy difference score (mean score at baseline assessment subtracted from mean score at post-assessment). The specified model equations are as follows:

$$GSE_{i}^{diff}=b_{0}+b_{1}GSE_{i}^{pre}+b_{2}group_{i}+b_{3}GSE_{i}THS_{i}+b_{4}\;GSE_{i}BHS_{i}+$$

$$b_{5}GSE_{i}STAI-Trait_{i}+b_{6}GSE_{i}STAI-State_{i}+b_{7}GSE_{i}Panas-Positive_{i}+$$

$$b_{8}GSE_{i}Panas-Negative+b_{9}GSE_{i}PSS_{i}+b_{10}GSE_{i}BDI_{i}$$

### Power analysis and sample size

An a priori power analysis was conducted for linear mixed models using the sjstats package in R. For alpha 0.05, power 80%, and an effect size of *d* = 0.6., the required sample size was 94 participants, which is in line with our recent review summarizing findings from mHealth ecological momentary intervention studies [72]).

## Results

### Sample characteristics

Table 1 summarizes the demographic and clinical variables. There were no differences between the groups in key demographic and clinical characteristics (all *p* ≥ .05).

### Effects of the digital self-efficacy training

**Fig 3:**
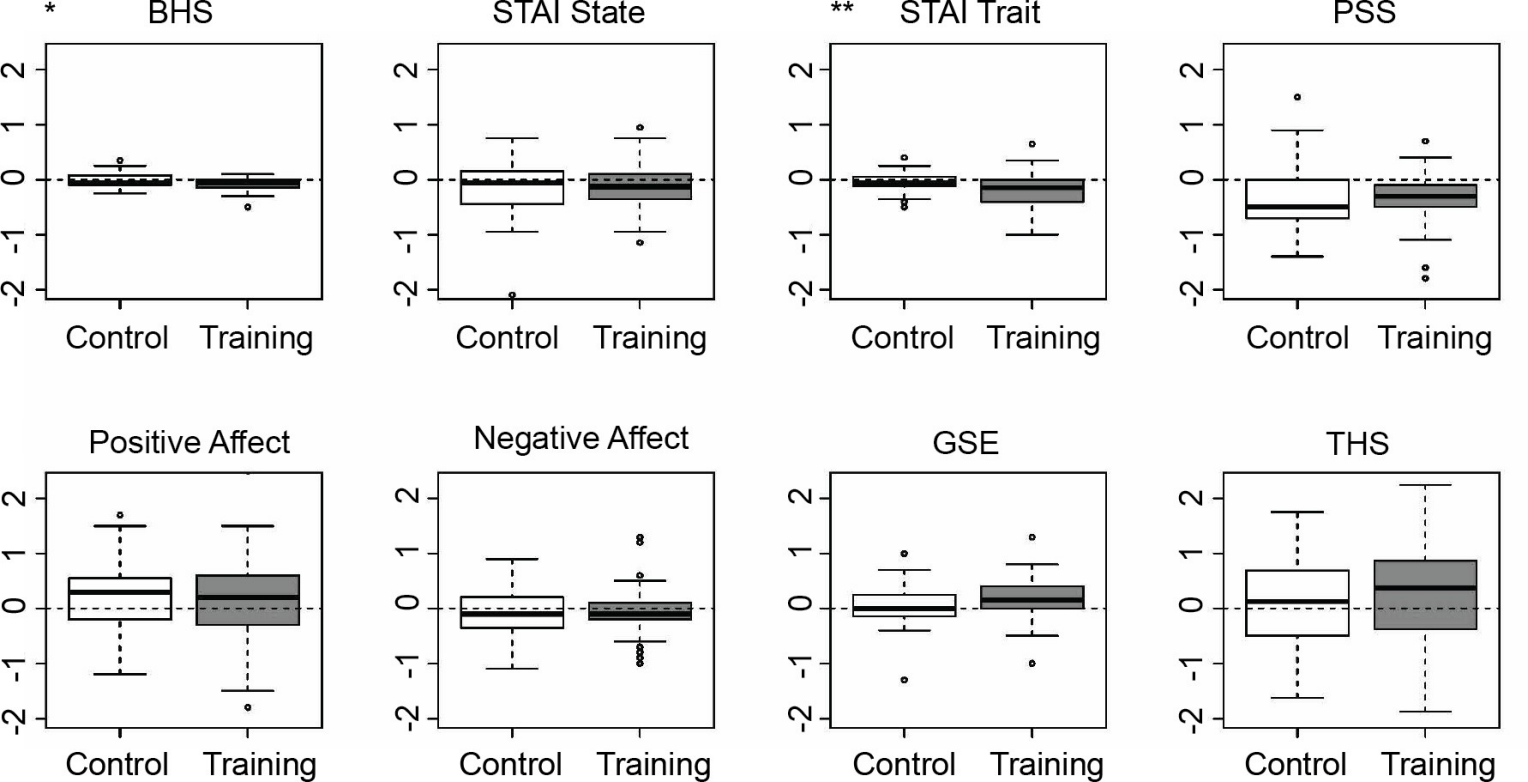
Pre- to post self-efficacy training versus control: Changes in outcomes. Changes were significant in BHS (*p* < 0.05) and STAI Trait (*p* < 0.01); BHS = Beck Hopelessness Scale, STAI State = State-Trait Anxiety Inventory (state subscale), STAI Trait = State-Trait Anxiety Inventory (trait subscale), PSS = Perceived Stress Scale, Positive Affect = Positive and Negative Affect Scale (positive affect subscale), Negative Affect = Positive and Negative Affect Scale (negative affect subscale), GSE = General Self-Efficacy Scale, THS = Trait Hope Scale; error bars represent the standard deviation.

As hypothesized, we observed a significant effect of the self-efficacy training on changes in hopelessness, *b* = −0.06, *SE* = 0.03, *p* = .02, *β* = 0.24 and trait anxiety, *b* = −0.18, *SE* = 0.06, *p* = .003, *β* = -0.30, whereby hopelessness and trait anxiety were lower after the training in the training group compared to the control group (Fig 3). No significant effects were detected for the other outcome variables, including self-efficacy. For detailed results, see S1 Table in the supporting information; complete data tables and codes are publicly available on https://osf.io/ucqjk/. No harm or unintended effects were reported in the feedback section after app participation.

### Predicting self-efficacy changes by baseline variables

**Fig 4:**
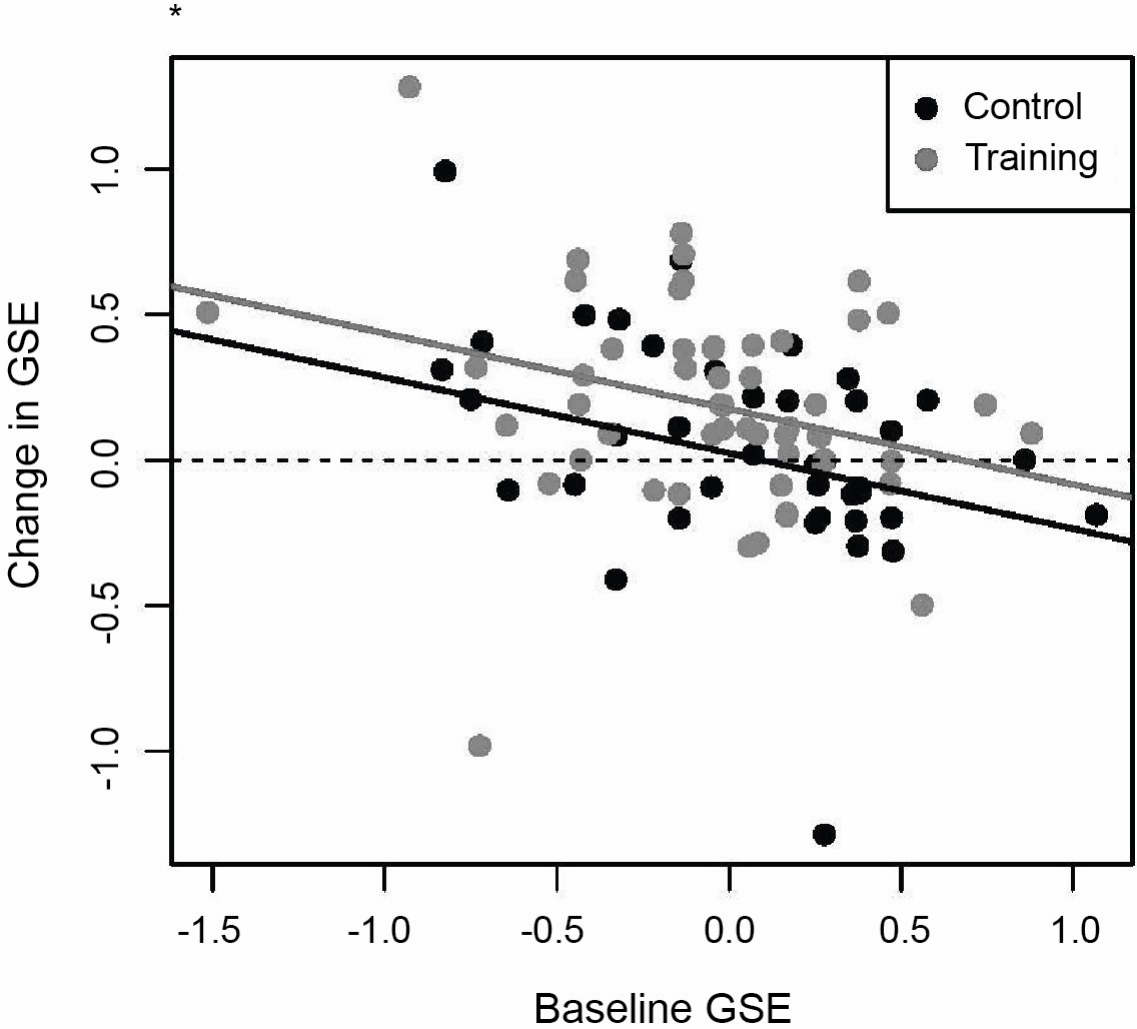
Changes in self-efficacy from pre- to post self-efficacy training controlled for baseline self-efficacy. Significant group difference in prediction of self-efficacy changes (*p* < 0.5); GSE = General Self-Efficacy Scale.

Lower baseline self-efficacy predicted greater self-efficacy changes in both groups, *b* = −0.26, *SE =* 0.09, *p =* .003, *β* = -0.31. This effect was significantly greater in the training group than in the control group, *b* = 0.15, *SE* = 0.07, *p* = .02, *β* = 0.21 (Fig 4). Higher baseline hopelessness and baseline self-efficacy were associated with an increase in self-efficacy in both groups, *b* = 2.68, *SE* = 0.64, *p* < .001, *β* = 0.97. Similarly, higher levels of baseline perceived stress and baseline self-efficacy predicted greater self-efficacy changes at post-assessment in both groups, *b* = 0.39, *SE* = 0.18, *p* = 0.03, *β* = 0.31. Higher baseline depression and baseline self-efficacy were associated with a smaller change in self-efficacy in both groups, *b* = −1.37, *SE* = 0.37, *p* < .001, *β* = 0.93. Likewise, high levels of baseline negative affect and baseline self-efficacy were associated with a smaller change in self-efficacy in both groups, *b* = −0.44, *SE* = 0.22, *p* = .049, *β* = -0.38. No significant effects were observed for the other variables. For detailed results, see S2 Table in the supporting information; complete data tables and codes are publicly available on https://osf.io/ucqjk/.

## Discussion

This study examined the effects of a one-week digital self-efficacy training implemented in the daily lives of university students experiencing elevated levels of stress during the first and second waves of the COVID-19 pandemic. In the training group, participants received psychoeducation and defined two autobiographical self-efficacy memories, which they were subsequently trained to repeatedly recall. No unintended side effects were reported.

Our study extends previous research on experimental self-efficacy induction and shows that participating in a self-efficacy training is associated with improvements in mental health outcomes. Compared to a control group engaging with the EMA only, the self-efficacy training group reported significantly reduced hopelessness and trait anxiety following training. There were no significant changes in other features, but the change in self-efficacy from pre- to post-training was significant in both groups when controlling for baseline self-efficacy with a significantly greater effect in the training group. We also found interaction effects of baseline self-efficacy with perceived stress and hopelessness, both of which were associated with greater changes in self-efficacy at post-assessment, and with baseline depression and negative affect, which were both associated with smaller changes in self-efficacy at post-assessment. The self-efficacy training did not lead to any deterioration in the psychological parameters.

In line with previous findings, we found significant effects of the self-efficacy training on hopelessness [17, 18] and trait anxiety [15]. Recalling autobiographical self-efficacy memories was only associated with significant direct effects on self-efficacy when controlling for baseline self-efficacy, indicating that participants with lower baseline self-efficacy benefitted more from the training than those who already had high levels of baseline self-efficacy. This leads to the assumption that individuals with lower baseline self-efficacy, e.g., people with mental health problems, may benefit more from the training. A methodological reason is also conceivable, in that using the GSE to measure self-efficacy might have been less suitable to capture the changes induced by the self-efficacy training thoroughly. The training aims to enhance self-efficacy by recalling autobiographical successes. However, participants’ chosen self-efficacy memory content could vary significantly and address different situation-specific self-efficacy aspects. While the GSE was selected to measure overall self-efficacy, it may not have ideally captured these specific aspects, despite evidence suggesting an interrelation between general and specific self-efficacy [73, 74]. For instance, the self-efficacy beliefs of individuals who have successfully managed various specific situations may generalize [75, 76]. While the literature introduces multiple concepts of self-efficacy, including distinctions like trait vs. state self-efficacy and general vs. domain- and task-specific self-efficacy, the evidence seems inconclusive. Thus, a common construct, possibly general self-efficacy, may be underlying different self-efficacy measures, including the GSE [74], aligning with the evaluation of the developers of the GSE [58]. Consequently, changes over time as a result of a one-week intervention may be more difficult to detect using any scale mainly capturing general aspects, particularly when measurement occurs immediately after the training. Using specific items based on the individually chosen themes, such as professional success, overcoming social challenges, or excelling in a sporting event, may be a future approach. In line with that, not all prior studies have reported post-self-efficacy intervention changes using the GSE [77]. Some formulated specific items based on Bandura’s definition [38] and others investigated related aspects following self-efficacy induction, such as distress tolerance, future thinking, social problem-solving, or perceived coping capability [77, 78]. Another approach could be applying the measure at a later time point.

### Limitations

This study is not without limitations. First, we did not implement longer-term follow-up measures, so we cannot elaborate on the long-term effects of the self-efficacy training. Second, we conducted the study during the first and second waves of the COVID-19 pandemic and detected elevated levels of stress, but we cannot make claims regarding causality. Third, although this training was developed based on existing knowledge of how self-efficacy can be enhanced using autobiographical self-efficacy memories, we cannot obviate that there might have been another active ingredient contributing to the observed effects. For example, engagement in EMA itself might have contributed to the observed effects, as also the control group reported changes in outcome variables. Indeed, mood monitoring has been found to have positive effects on mental health; for instance, it can reduce negative mood [79] and facilitate self-awareness [80]. However, we believe that this may have not affected the group differences in outcomes since the control group engaged in the same EMA, which might have reduced the potential factors. Fourth, our sample was homogeneous with respect to the level of education, sex, and origin, and replications with data from more heterogeneous samples, including other cultural contexts as well as mental health problems beyond increased stress levels are warranted. However, since this was the first implementation of our training, we found it important to first investigate it in a non-clinical population. Fifth, regarding the different number of participants in each group, we did not find significant group differences and therefore assume that the imbalance did not have any influence on the results. Sixth, we would like to mention that the study was underpowered since we examined 93 participants instead of the aimed 94 participants according to our power analysis. Seventh, due to the high number of participants not providing any data (76% of the participants that were not included in the analysis dropped out even before the baseline assessment took place), we did not include an intention-to-treat analysis, but only a per-protocol analysis. Lastly, this study was not registered prior to conduction, but retrospectively. Some of these limitations are shared by similar studies [81].

### Strengths and clinical implications

However, our study also has several strengths and clinical implications. We showed that a one-week digital self-efficacy training was well tolerated and may significantly improve hopelessness and trait anxiety in students, which are commonly reported symptoms in clinical populations, especially during challenging times such as the COVID-19 pandemic [5–7]. Although self-efficacy has previously been shown to be associated with hopelessness and trait anxiety, to the best of our knowledge, this is the first digitally administered self-efficacy training incorporated into participants’ daily lives that positively influences these psychological variables. Furthermore, the training significantly enhanced self-efficacy in individuals with low self-efficacy, which is known to be associated with mental health problems [82–86], which suggests that the effects of the self-efficacy training might be more pronounced in clinical populations. Finally, individuals with high levels of stress, hopelessness, and anxiety particularly benefitted from the training, indicating the relevance of screening for these aspects in the future to find out which individuals would benefit most from the training.

## Conclusions

Taken together, the self-efficacy training had significant positive effects on relevant mental health outcomes in a non-clinical population. Results should now be replicated in more heterogeneous samples and settings, especially in clinical populations where we expect the positive effects to be greater. Specifically, the self-efficacy training could be beneficial in particular situations, such as prior to specialized psychotherapy, possibly to bridge waiting times to reduce potential demotivation while awaiting treatment [87]. In such patients, engaging in the self-efficacy training might build key capacities that may not only alleviate symptom burden and decrease hopelessness but also facilitate subsequent therapy. This is supported by studies showing that higher levels of pre-treatment self-efficacy and increased self-efficacy in the course of treatment are predictors of the therapeutic process and improve therapy outcomes (2). Indeed, self-efficacy has been associated with improved therapy motivation [83], treatment adherence, and outcomes [88, 89]. Future work could thus implement such training in clinical populations and investigate long-term effects, which could yield further insights into the effects and benefits of digital self-efficacy training.

## Supporting information

S1 Table Efficacy of the self-efficacy training: Results of the multivariate linear model for group and compliance as predictors. BHS = Beck Hopelessness Scale, STAI State = State-Trait Anxiety Inventory (state subscale), STAI Trait = State-Trait Anxiety Inventory (trait subscale), PSS = Perceived Stress Scale, PANAS Pos = Positive and Negative Affect Schedule (positive affect subscale), PANAS Neg = Positive and Negative Affect Schedule (negative affect subscale), GSE = General Self-Efficacy Scale, THS = Trait Hope Scale.
(DOCX)

S2 Table Prediction of self-efficacy outcome by baseline variables: Results of the multiple linear regression model on difference score of self-efficacy. GSE = General Self-Efficacy Scale, THS = Trait Hope Scale, BHS = Beck Hopelessness Scale, STAI State = State-Trait Anxiety Inventory (state subscale), STAI Trait = State-Trait Anxiety Inventory (trait subscale), PANAS Pos = Positive and Negative Affect Schedule (positive affect subscale), PANAS Neg = Positive and Negative Affect Schedule (negative affect subscale), PSS = Perceived Stress Scale, BDI-II = Beck Depression Inventory II.
(DOCX)

S1 File Histograms of the change scores. (PDF)

S2 File Study protocol. (PDF)

S3 File Summary of deviations to the study protocol submitted to the ethics boards. (DOCX)

S1 Checklist CONSORT 2010 checklist of information to include when reporting a randomised trial*. (DOC)
