# Supplementary material for: Effects of a digital self-efficacy training in stressed university students: A randomized controlled trial
Source: PLoS One. 2024 Oct 31;19(10):e0305103. doi: 10.1371/journal.pone.0305103 (PMC11527301; doi:10.1371/journal.pone.0305103)
Supplement: S1 Table — BHS = Beck Hopelessness Scale, STAI State = State-Trait Anxiety Inventory (state subscale), STAI Trait = State-Trait Anxiety Inventory (trait subscale), PSS = Perceived Stress Scale, PANAS Pos = Positive and Negative Affect Schedule (positive affect subscale), PANAS Neg = Positive and Negative Affect Schedule (negative affect subscale), GSE = General Self-Efficacy Scale, THS = Trait Hope Scale. (DOCX) [file pone.0305103.s001.docx]

**Table S1**

**Results: Effects of the Digital Self-Efficacy Training**

| Difference score | Predictor | Estimate | Standard Error | Z Value | P Value | Beta Coefficient |
| --- | --- | --- | --- | --- | --- | --- |
| Hopelessness (BHS) | Group | -0.06 | 0.03 | -2.31 | 0.02 | -0.24 |
| State Anxiety (STAI State) |  | 0.05 | 0.01 | 0.51 | 0.61 | 0.05 |
| Trait Anxiety (STAI Trait) |  | -0.18 | 0.06 | -3.01 | 0.003 | -0.30 |
| Perceived Stress (PSS) |  | -0.02 | 0.11 | -0.19 | 0.86 | -0.02 |
| Positive Affect (PANAS Pos) |  | -0.11 | 0.15 | -0.72 | 0.47 | -0.08 |
| Negative Affect (PANAS Neg) |  | -0.009 | 0.11 | -0.09 | 0.93 | -0.01 |
| Self-Efficacy (GSE) |  | 0.13 | 0.08 | 1.65 | 0.10 | 0.17 |
| Hope (THS) |  | 0.16 | 0.25 | 0.65 | 0.52 | 0.07 |
| Hopelessness (BHS) | Compliance | -0.02 | 0.06 | -0.25 | 0.81 | -0.03 |
| State Anxiety (STAI State) |  | 0.25 | 0.21 | 1.19 | 0.24 | 0.12 |
| Trait Anxiety (STAI Trait) |  | 0.05 | 0.13 | 0.38 | 0.71 | 0.04 |
| Perceived Stress (PSS) |  | -0.34 | 0.24 | -1.40 | 0.16 | -0.15 |
| Positive Affect (PANAS Pos) |  | 0.15 | 0.34 | 0.43 | 0.67 | 0.05 |
| Negative Affect (PANAS Neg) |  | 0.59 | 0.24 | 2.50 | 0.01 | 0.25 |
| Self-Efficacy (GSE) |  | -0.23 | 0.17 | -1.38 | 0.17 | -014 |
| Hope (THS) |  | -0.21 | 0.55 | -0.37 | 0.71 | -0.04 |
