# Supplementary material for: Effects of a digital self-efficacy training in stressed university students: A randomized controlled trial
Source: PLoS One. 2024 Oct 31;19(10):e0305103. doi: 10.1371/journal.pone.0305103 (PMC11527301; doi:10.1371/journal.pone.0305103)
Supplement: S2 Table — GSE = General Self-Efficacy Scale, THS = Trait Hope Scale, BHS = Beck Hopelessness Scale, STAI State = State-Trait Anxiety Inventory (state subscale), STAI Trait = State-Trait Anxiety Inventory (trait subscale), PANAS Pos = Positive and Negative Affect Schedule (positive affect subscale), PANAS Neg = Positive and Negative Affect Schedule (negative affect subscale), PSS = Perceived Stress Scale, BDI-II = Beck Depression Inventory II. (DOCX) [file pone.0305103.s002.docx]

**Table S2**

**Results: Predicting Self-Efficacy by Baseline Variables**

| Difference Score | Predictor | Estimate | Standard Error | Z Value | P Value | Beta Coefficient |
| --- | --- | --- | --- | --- | --- | --- |
| Self-Efficacy (GSE) | Self-Efficacy (GSE) | -0.26 | 0.09 | -2.98 | 0.003 | -0.31 |
| Self-Efficacy (GSE) | Group | 0.15 | 0.07 | 2.28 | 0.02 | 0.21 |
| Self-Efficacy (GSE) | Self-Efficacy (GSE) / Trait Hope (THS) | 0.15 | 0.0 | 1.71 | 0.09 | 0.24 |
| Self-Efficacy (GSE) | Self-Efficacy (GSE) / Hopelessness (BHS) | 2.68 | 0.64 | 4.17 | 0.000 | 0.97 |
| Self-Efficacy (GSE) | Self-Efficacy (GSE) / State Anxiety (STAI State) | 0.20 | 0.22 | 0.90 | 0.37 | 0.14 |
| Self-Efficacy (GSE) | Self-Efficacy (GSE) / Trait Anxiety (STAI Trait) | 0.15 | 0.28 | 0.53 | 0.59 | 0.11 |
| Self-Efficacy (GSE) | Self-Efficacy (GSE) / Positive Affect (PANAS Pos) | 0.06 | 0.17 | 0.35 | 0.72 | 0.06 |
| Self-Efficacy (GSE) | Self-Efficacy (GSE) / Negative Affect (PANAS Neg) | -0.44 | 0.22 | -1.97 | 0.049 | -0.38 |
| Self-Efficacy (GSE) | Self-Efficacy (GSE) / Perceived Stress (PSS) | 0.39 | 0.18 | 2.16 | 0.03 | 0.31 |
| Self-Efficacy (GSE) | Self-Efficacy (GSE) / Depression (BDI-II) | -1.37 | 0.37 | -3.72 | 0.000 | -0.93 |
