# Supplementary figures and images for: Effects of a digital self-efficacy training in stressed university students: A randomized controlled trial
Source: PLoS One. 2024 Oct 31;19(10):e0305103. doi: 10.1371/journal.pone.0305103 (PMC11527301; doi:10.1371/journal.pone.0305103)

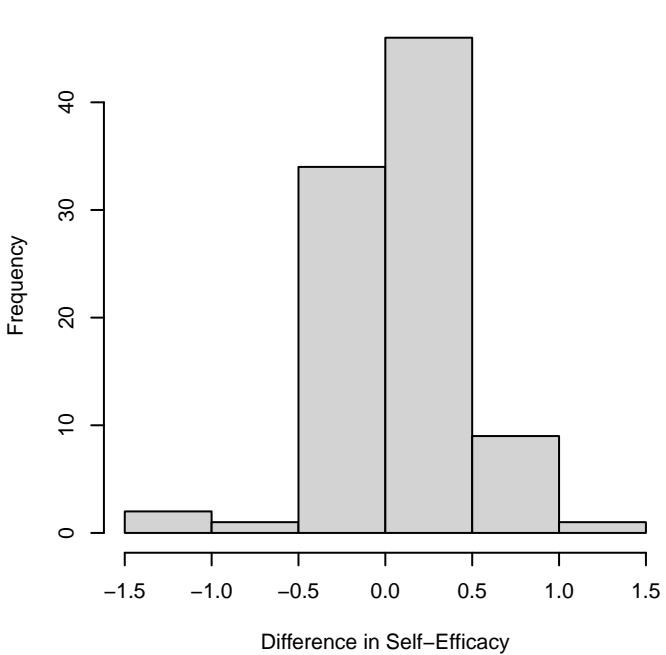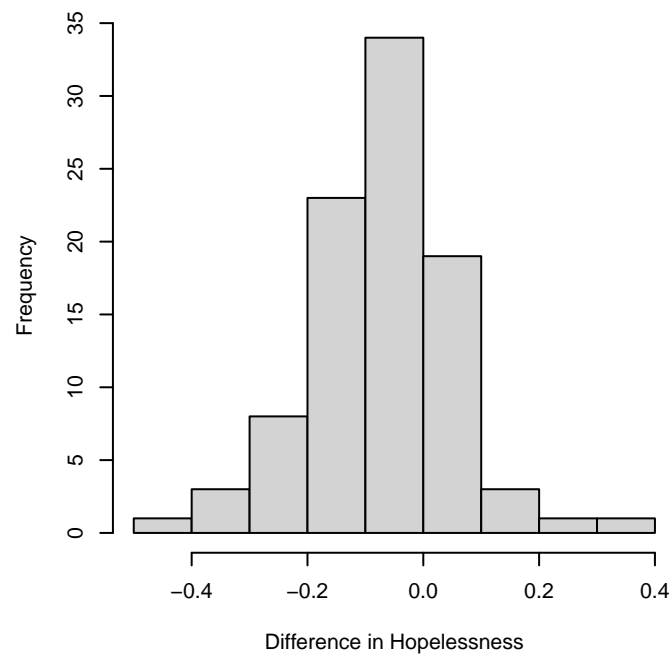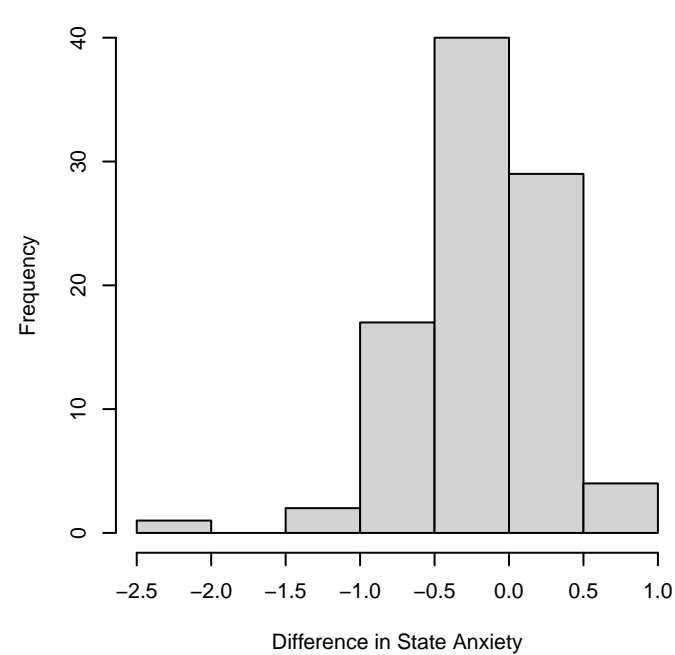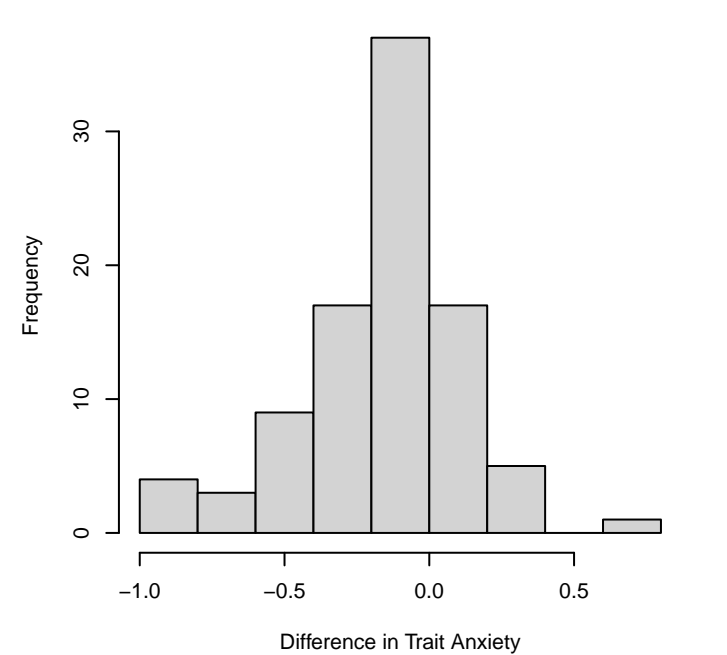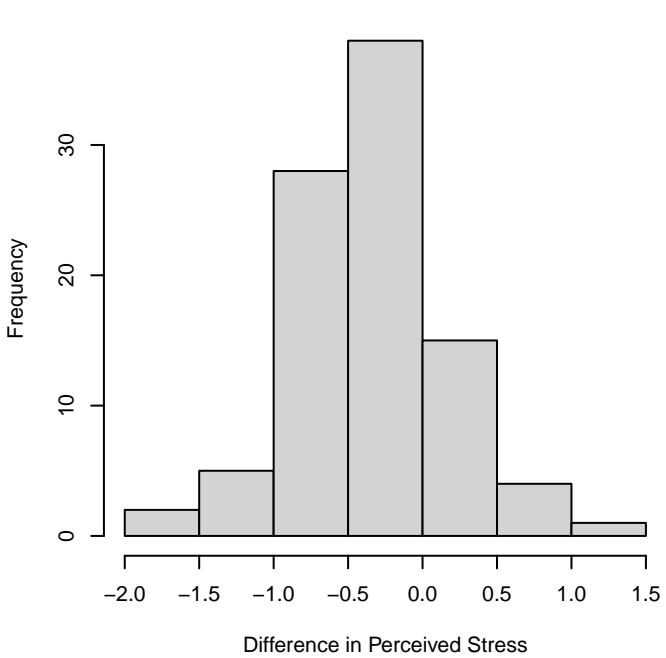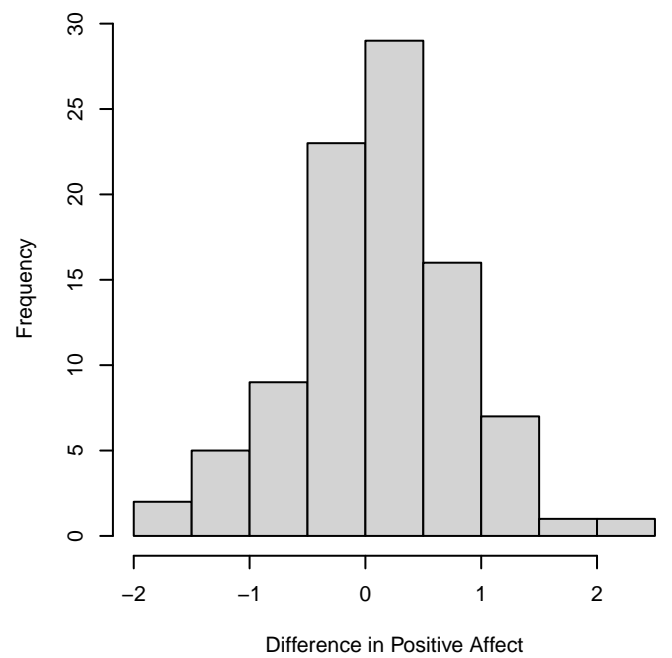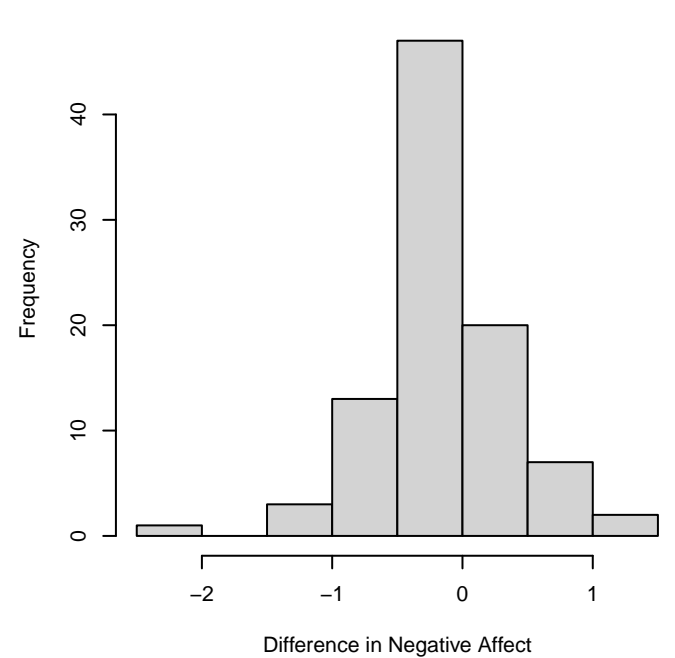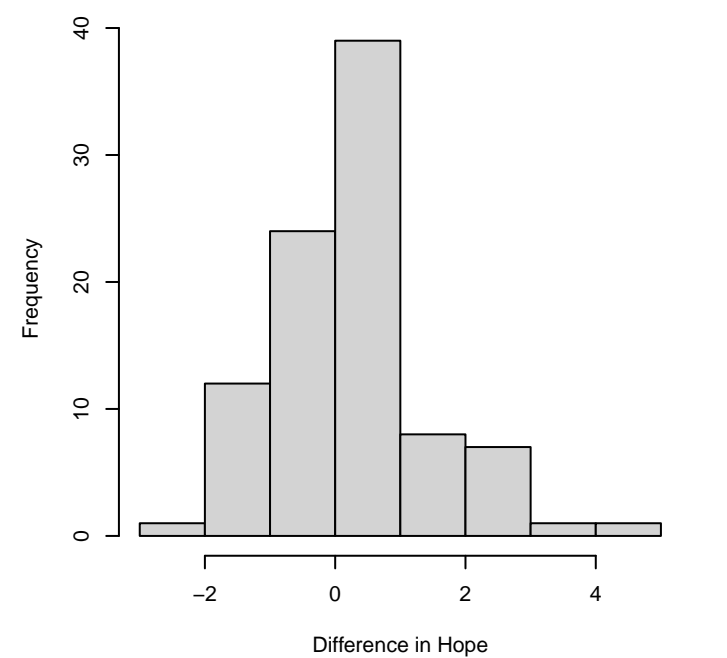

Supplement: S1 File — (PDF) [file pone.0305103.s003.pdf]
