# Supplementary material for: Effects of a digital self-efficacy training in stressed university students: A randomized controlled trial
Source: PLoS One. 2024 Oct 31;19(10):e0305103. doi: 10.1371/journal.pone.0305103 (PMC11527301; doi:10.1371/journal.pone.0305103)
Supplement: S2 File — (PDF) [file pone.0305103.s004.pdf]

Study Protocol including procedures & material

SELF-EFFICACY STUDY

JUDITH ROHDE ET AL.

04/2020

TABLE OF CONTENTS

INTRODUCTION **3**
GENERAL NOTE 3
AIM OF THE STUDY 3
STUDY DESIGN 4
QUESTIONNAIRES 4
PARTICIPANTS 5
Inclusion & exclusion criteria *5*
Screening *5*
Recruitment *6*
BASELINE **6**
IDS AND APP ASSIGMENT 7
FOLLOW-UP **8**
END OF STUDY **8**
COMPENSATION 8
DATA 8
TECHNICALITIES **9**
SETTING UP NEW RAS 9
DATA EXPORT FOR BACK UP 9
STUDY MONEY 10
APPENDIX **11**
PERCEIVED STRESS SCALE (PSS), GERMAN VERSION 11

INTRODUCTION

GENERAL NOTE

This protocol provides a summary of the SeApp study and information on the different stages of the study. Based on it, we will apply for the ethics approval. Later changes to this protocol will be collected in the study folder.

AIM OF THE STUDY

The aim of the project is to test a recently developed, smartphone-based self-efficacy Ecological Momentary Intervention (EMI) that focuses on psychoeducation and daily recall of self-efficacy memories. EMIs are mostly smartphone-based applications that deliver interventions to people while being engaged in their daily life activities. This app harnesses the power of self-efficacy autobiographical memories, is based on theoretical and empirical findings, and an extension of our previous laboratory-based interventions. We want to test its feasibility and to investigate how daily self-efficacy can be enhanced and perceived stress reduced by a daily memory-based training via smartphone app. Specifically, we want to investigate the effects of the training from baseline to post intervention (change of main outcomes general self-efficacy, anxiety, positive and negative affect, hope, hopelessness, stress). We are also interested in collecting Ecological Momentary Assessment (EMA) data and exploring relations, e.g., to baseline data.

The SeApp study aims to test the EMI in healthy students of health care and non-health care majors in the context of COVID-19 and other stressful events (e.g., exam period). It will be delivered daily for one week and it will be combined with a daily assessment on different parameters (EMA). Using EMA, we will monitor mood as well as social and virtual contacts. We will compare the interventions' effect to a control group, where the Ecological Momentary Assessment is applied only.

Study Design

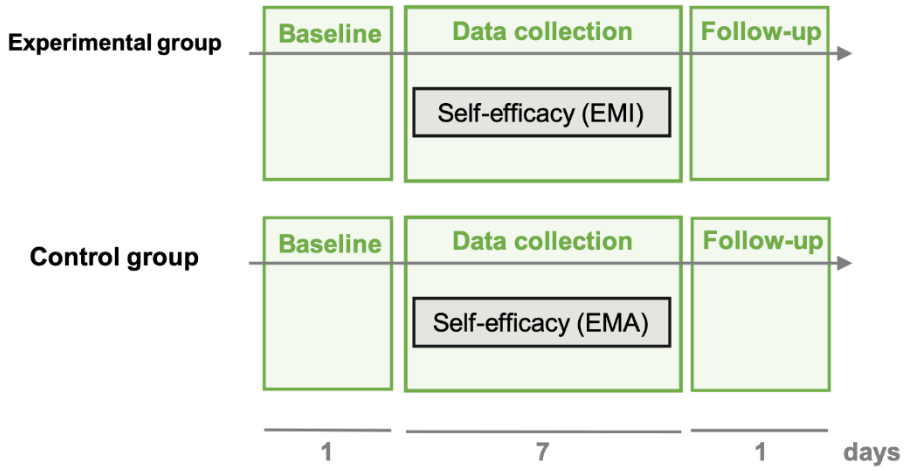

For this RCT, we will screen student participants. If participants have a score of ≥ 13 on the Perceived Stress Scale (PSS; see appendix), they are eligible for the study if other inclusion criteria are fulfilled as well as and if they do not state to have a psychiatric diagnosis or history. Then, participants will be randomly assigned to a training (self-efficacy EMI and EMA) and control group (EMA only).

Questionnaires

	Screening	Baseline	Follow up
Perceived Stress Scale (PSS)	x		x
Life Events Questionnaire		x	
Childhood Trauma Questionnaire		x	
Beck Depression Inventory-II (BDI)		x	x
State-Trait Anxiety Inventory (STAI)		x	x
Cognitive Emotion Regulation (CERQ)		x	x
Emotion Regulation Scale (ERS)		x	x
Positive and Negative Affect Schedule (PANAS)		x	x
Patient Health Questionnaire (PHQ)		x	x
Pittsburgh Sleep Quality Inventory (PSQ)		x	x
Interpersonal Needs Questionnaire (INQ)		x	x
Beck Hopelessness Scale (BHS)		x	x
Generalized Self-Efficacy Scale (GSE)		x	x
The Trait Hope Scale		x	x

UCLA Loneliness Scale		x	x
Research Experience Questionnaire (REQ)			x
Mobile Application Rating Scale (uMARS)			x

PARTICIPANTS

Eighty healthy students with heightened levels of perceived stress who will be screened for perceived stress (perceived stress scale, PSS, cutoff 13) will be included. Participants will be randomly assigned to a training (self-efficacy Ecological Momentary Intervention (EMI) + Ecological Momentary Assessment (EMA)) or a control condition completing EMA only. They will be asked to complete a baseline assessment. Participants randomized to the self-efficacy training group will be invited to watch a 4 min online video that will give general information on self-efficacy and the SeApp. They will also be asked to select two autobiographical self-efficacy memories. The app training will then last seven consecutive days and the app will send ten prompts per day with either the EMA or the combined EMI + EMA (3/10) resulting in a total 70 assessments resp. 21 training sessions. After using the self-efficacy app or EMA, participants will again complete an assessment (follow-up one week after assignment).

INCLUSION & EXCLUSION CRITERIA

**Inclusion criteria**	**Exclusion criteria**
1. Value on Perceived Stress Scale ≥ 13<br>2. Student at Swiss university<br>3. Owner of a smartphone<br>4. Speaking German fluently<br>**5.** 18-29 years old	1. Psychiatric history

SCREENING

We will recruit on social media platforms and through university platforms and mailing lists. The study information includes the informed consent form and a link to UniPark where students fill in the screening questionnaire, which includes the Perceived Stress Scale in German (PSS, for more details see in the appendix *Perceived Stress Scale*).

RECRUITMENT

Once potential participants have filled in the PSS on UniPark, we decide based on their score the following procedure.
How to:

- Go to UniPark
- Login with your own credentials
- Check for newly filled in PSS questionnaires by exporting data → select only for the time frame of the previous day
- then select «*Export*» in the right column. Now «*Export von*»… «*Ergebnisdaten (alle Angaben, Formate z. B. SPSS, CSV, Fixed Format, Microsoft Excel...)*», select «*weiter*», select as Dateiformat «*XLS (binäres Excel Format)*» (leave the rest (z.B. Dateikomprimierung) as it is), select «*Exportieren*». On the next page choose «*Datei auf den lokalen PC kopieren*»
- also download the current assignment file (screening_assignnment-date) from the server and fill out the columns and fill in the corresponding cells → Codes for the variables used in the exported Excelfile can be checked on UniPark (Fragebogen-Editor → Codebook)
- Calculate the overall PSS sum score by summing it up (be aware of the polarity of the items, sum up the items 1, 2, 3, 6, 9, 10 and reverse the scores for questions 4, 5, 7, and 8 to the PSS score

**PSS < 13**	**PSS ≥ 13**
Send the email "*if students are not eligible due to a too low stress level"* to the student	The student is eligible

- Participants with a psychiatric diagnosis are not allowed to participate in the study. In this case send the email "*if the students do not meet the inclusion or exclusion criteria*"
- if the participants have been assigned, the file should be uploaded to the server again, so that the participants can be contacted by the respective study personnel

BASELINE

1. Check the screening_assignment.xlsx file on the server for all participants that have been assigned to the study

2. Email participants the a) personal code and b) link to UniPark for the baseline questionnaire
3. Baseline assessment needs to be completed within one week. We will send out one reminder email.

IDs and App Assigment

Important: IDs do not start with 0 but with 100, which means participant who used for example 001 for the baseline questionnaire will get the ID 101

1. After they fill in the UniPark baseline questionnaire
2. Send the email according to group assignments
3. Invite them to SEMA3 platform:

    1. Log in
    2. Click on “participants”
    3. Click on “invite participants”
    4. In “name” put their personal code
    5. In “mail” put email
    6. Click on “verify”
    7. Export participant data
    8. Set up “start date” according to the start date
    9. Click on “send invites”

Then….

1. Click on “Edit” on the right side
2. Change participant state: check if this is active
3. Change Participant Charts Preview: check if this is stopped
4. Change Participant Permission: check if this is user
5. Surveys and schedules - keep the schedules <u>default</u>

FOLLOW-UP

On day 8, email the participant further instructions for the follow-up assessment. This assessment needs to be completed within one week as well. We will send out one reminder email.

END OF STUDY

1. Check in UniPark if follow-up questionnaires were filled in completely.
2. Enable mood charts in SEMA3
    a. Log in
    b. Click on “participants”
    c. Click on “Edit” on the right side
    d. Change participant state to STOPPED
    e. Change Participant Charts Preview: ACTIVE
3. Send participants the below email with further instructions for the study’s end.

COMPENSATION

Participants are reimbursed with up to CHF 60 or 3 study credits. For filling in the baseline and followup questionnaires, participants receive CHF 40. If their compliance for the EMA part was more than 60%, they receive an additional CHF 20. Was their compliance lower than 60% they receive no additional payment. The money will be transferred either via TWINT or bank transfer.

DATA

Collected data will be archived after the completion of the study for a period of 10 years. The data and the decoding list will be archived separately. Only researchers who are involved in the study will have access to the data. Personal data of participants (email address) needed to send email invitations to questionnaires will irreversibly deleted at the end of the study. After completion of data collection, data will be analyzed using multilevel modeling, investigating training effects.

TECHNICALITIES

SETTING UP NEW RAS

Beforehand

- Fill in the «Formular Meldung von Mitarbeitenden ohne Lohnzahlung PUK» and send it to Dorjee Bargemann (dorjee.bargemann@puk.zh.ch)

Upon start

- PUK-equipment:
    - pick up keys
    - go by IT services to get account information
    - go by the cafeteria to pick up your Mitarbeiter-Karte
- Computer programs
    - MCC = the electronic paten record data base
    - RAP = the electronic calendar for staff and patients
    - PEP = the electronic system to save your working hours
    - Login with the details you got from IT services (extension: 6060) and change password
- Add printer (BLIMFP 018 at the secretary)
    - Go to intranet for instructions: choose "dokumente" → "suchbegriff" → "drucker" → "drucker hinzufugen"

To access your PUK email account from home go to https://mail.pukzh.ch using your login credentials

- Other
    - Study data will be saved on the server
    - Study email address: seapp@psychologie.uzh.ch

DATA EXPORT FOR BACK UP

- Proceed as follows to export screening, baseline and follow-up data (Excel and CSV). This example shows the back up of screening data (login and screenshots; see below)
    - Go to UniPark
    - Login with your own credentials
    - Now select study on the first screen that appears when you log in to UniPark

- then select «*Export*» in the right column. Now «*Export von*»… «*Ergebnisdaten (alle Angaben, Formate z. B. SPSS, CSV, Fixed Format, Microsoft Excel...)*», select «*weiter*», select as Dateiformat «*CSV (Spalten sind durch Trennzeichen getrennt, keine Label)*» (leave the rest (z.B. Dateikomprimierung) as it is), select «*Exportieren*». On the next page choose «*Datei auf den lokalen PC kopieren*»

- now do the same procedure to download the CSV: select «*Export*» again in the right column. Now «*Export von*»… «*Ergebnisdaten (alle Angaben, Formate z. B. SPSS, CSV, Fixed Format, Microsoft Excel...)*», select «*weiter*», select as Dateiformat «*XLS (binäres Excel Format)*» (leave the rest (z.B. Dateikomprimierung) as it is), select «*Exportieren*». On the next page choose «*Datei auf den lokalen PC kopieren*»
- follow the same procedure for baseline data and for follow-up data
- for the next step please go to the server and upload the files (CSV and Excel) you just downloaded from UniPark in the respective folders "t0 - Screening", "t1 - baseline" and "t2 - followup”. Name the files like the previous ones (with the current date)

STUDY MONEY

Steps that have to be taken beforehand so that money is present for participants:

1. Send Dorjee information about how much money is needed for the next couple of months
2. Dorjee prepares the necessary document that Birgit has to sign
3. Pick up that document and bring it to

Hauptkasse
Künstlergasse 17
8001 Zürich
Tel.: 044 634 41 16

Open:
Mon- Fri
09.00 – 11.00 and 14.00 – 16.00

Here we can pick up the cash. The remainder of the cash & the proofs of participants that they have received the money should be brought back to the Hauptkasse before a particular date. The next end-date for this, is the **June 30, 2020**.

APPENDIX

PERCEIVED STRESS SCALE (PSS), GERMAN VERSION

	0 = Nie	1 = Fast nie	2 = Manch-mal	3 = Ziemlich oft	4 = Sehr oft
1. Wie oft hatten Sie sich im letzten Monat darüber aufgeregt, dass etwas völlig Unerwartetes eingetreten ist?					
2. Wie oft hatten Sie im letzten Monat das Gefühl, wichtige Dinge in Ihrem Leben nicht beeinflussen zu können?					
3. Wie oft hatten Sie sich im letzten Monat nervös und „gestresst“ gefühlt?					
4. Wie oft hatten Sie sich im letzten Monat sicher im Umgang mit persönlichen Aufgaben und Problemen gefühlt?					
5. Wie oft hatten Sie im letzten Monat das Gefühl, dass sich die Dinge nach Ihren Vorstellungen entwickeln?					
6. Wie oft hatten Sie im letzten Monat das Gefühl, mit all den anstehenden Aufgaben und Problemen nicht richtig umgehen zu können?					
7. Wie oft hatten Sie im letzten Monat das Gefühl, mit Ärger in Ihrem Leben klarzukommen?					
8. Wie oft hatten Sie im letzten Monat das Gefühl, alles im Griff zu haben?					
9. Wie oft hatten Sie sich im letzten Monat darüber geärgert, wichtige Dinge nicht beeinflussen zu können?					
10. Wie oft hatten Sie im letzten Monat das Gefühl, dass sich die Probleme so aufgestaut haben, dass Sie diese nicht mehr bewältigen können?					
