# Supplementary material for: Effects of a digital self-efficacy training in stressed university students: A randomized controlled trial
Source: PLoS One. 2024 Oct 31;19(10):e0305103. doi: 10.1371/journal.pone.0305103 (PMC11527301; doi:10.1371/journal.pone.0305103)
Supplement: S3 File — (DOCX) [file pone.0305103.s005.docx]

**Summary of deviations to the study protocol submitted to the ethics boards**

Following piloting and the first study assessments, some adaptations were made to the original and IRB approved ethics form (dated April 15, 2020). Most of these changes did, according to the IRB’s standards and operating procedures, not require a full IRB reassessment and were silently approved. The following changes were made to the protocol:

1. The number of participants to be recruited into both conditions was increased from 80 to 94 according to an adapted power analysis.
2. We did not collect the one month follow up data.
3. The total number of questionnaires was reduced; questionnaires on hope and affect were added.
4. To ensure blinding of the participants to group assignment, reference to a self-efficacy training was omitted in the participant’s study information.
